# Polarization-dispersive imaging spectrometer for scattering circular dichroism spectroscopy of single chiral nanostructures

**DOI:** 10.1038/s41377-022-00755-2

**Published:** 2022-03-18

**Authors:** Shuang Zhou, Jie Bian, Peng Chen, Mo Xie, Jie Chao, Wei Hu, Yanqing Lu, Weihua Zhang

**Affiliations:** 1 College of Engineering and Applied Sciences, MOE Key Laboratory of Intelligent Optical Sensing and Manipulation, Nanjing university, Nanjing, 210023 China; 2 State Key Laboratory of Analytical Chemistry for Life Science, and Jiangsu Key Laboratory of Artificial Functional Materials, Nanjing University, Nanjing, 210093 China; 3 Key Laboratory for Organic Electronics and Information Displays (KLOEID) & Jiangsu Key Laboratory for Biosensors, Institute of Advanced Materials (IAM), National Synergetic Innovation Center for Advanced Materials (SICAM), Nanjing University of Posts and Telecommunications, 9 Wenyuan Road, Nanjing, 210023 China

**Keywords:** Circular dichroism, Optical materials and structures

## Abstract

Circular dichroism spectroscopy is one of the most important tools in nanoscopic chiroptics. However, there is lack of simple, fast and reliable method for measuring the circular dichroism responses of single nanostructures. To tackle this issue, we report a polarization-dispersive imaging spectrometer which is capable of measuring the scattering circular dichroism response of a single chiral nanostructure with a single shot. Using this technique, we studied the scattering circular dichroism spectra of a model system, the vertically coupled plasmonic nanorod pair. Both experimental and theoretical results indicate that the polarization-dispersive spectrometer measures the imaginary part of nonlocal susceptibility of the structure. We further applied the technique to 3-dimensional Au nanorod structures assembled on DNA origami templates together with correlated scanning electron microscopic measurements. Rich chiroptical phenomena were unveiled at the single nanostructure level.

## Abstract (web summary)

Polarization-dispersive imaging spectrometer enables simple, fast and reliable circular dichroism measurement for single chiral nanostructures.

## Graphical abstract

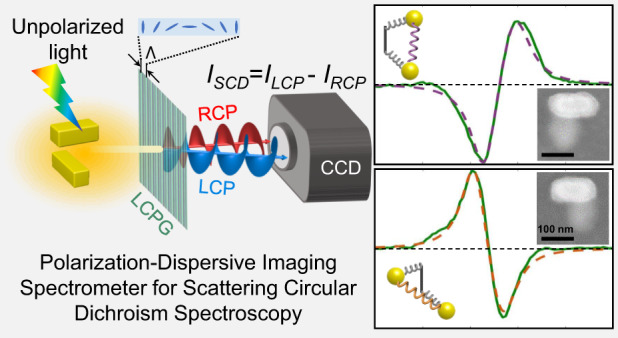

## Introduction

Circular dichroism (CD) spectroscopy is the most widely used optical tool for studying the 3D conformation of molecules and nanostructure, such as chirality of molecules^1,2^, conformation of proteins^3–6^ and chiroptical properties of nanostructures^7,8^. Today, with the rise of the field of chiral plasmonics, various chiral nanostructures^9–12^, even kinetic ones^13–15^, have been created. How to collect the CD spectrum of a single nanostructure in real-time and further retrieve its 3D structural information have become one of the central challenges of the field. However, to obtain the CD response, one currently needs to modulate the polarization state of the illumination and collect the spectra at different polarization states in separated steps^16–18^. This multi-step scheme makes the conventional CD spectral measurements very slow and consequently not applicable for continuous real-time measurements.

Interestingly, polarization-dependent dispersion effects were recently reported using metasurfaces^19–23^ and patterned liquid crystals^24,25^. It leads to the new possibility of building polarization-dispersive CD spectrometers similar to the wavelength-dispersive spectrometer, which has significantly simplified the spectral measurements. Among the polarization-dispersive devices, a particularly interesting one is the liquid crystal polarization grating (LCPG)^26,27^. It splits the left-hand circularly polarized (LCP) and right-hand circularly polarized (RCP) components of a beam into different diffraction orders^28,29^. One can therefore perform CD measurements by putting a LCPG in front of the camera in a dark-field microscope system and collecting the resulted spectral image, similar to the widely used wavelength-dispersive spectrometer for single nanoparticle spectroscopy developed by Sönnichsen et al.^30^.

More interestingly, it was reported that the CD responses of vertically coupled plasmonic nanorods can be explicitly described using the Born–Kuhn model^8^. This provides us a theoretical framework to understand the CD spectra by the new polarization-dispersive-device-based technique. Inspired by the above progresses, in this work, we develop a simple LCPG-based polarization-dispersive imaging spectrometer for the scattering CD (SCD) spectroscopy of single nanostructures, and further study its data interpretation in theory. The goal is to develop a simple, powerful and reliable spectroscopic tool for investigating 3D chiral nanostructures.

## Results

### LCPG-based polarization-dispersive imaging spectrometer

**Fig. 1:**
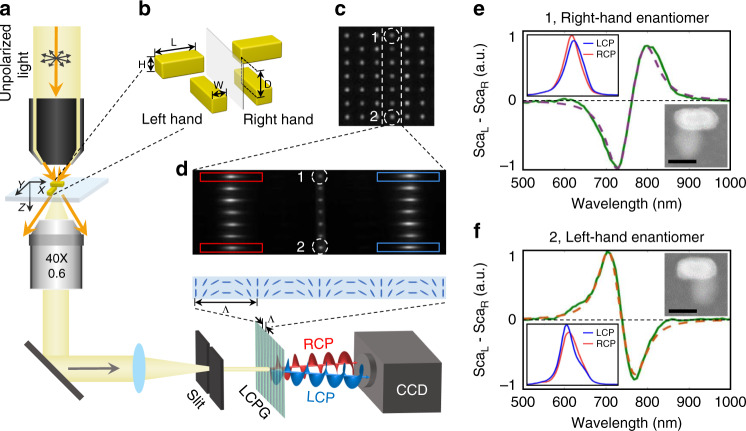
Polarization-dispersive imaging spectrometer for single nanostructure SCD spectroscopy. **a** Schematic drawing of the LCPG-based polarization-dispersive spectrometer. The inset illustrates the director distribution of the liquid crystal in the grating. **b** Structure of the vertically coupled nanorod pairs used in this work. **c** Dark-field image of an array of vertically coupled nanorod pairs with different geometrical parameters. The period of the array is 3 μm. **d** Spectral image of array of nanorod pairs labeled in (**c**). The RCP and LCP components are labeled with red and blue boxes, respectively. **e**, **f** are the SCD spectra of sample 1 and 2 in (**c**), respectively. The insets show the correlated SEM image of samples, as well as the left-hand and right-hand circularly polarized components of the scattered light of sample labeled in (**d**). Scale bar: 100 nm

Figure 1a is the schematic drawing of the SCD spectroscopy system. It consists of a standard dark-field microscope and a home-made imaging spectrometer, in which a LCPG (grating period *Λ* = 18 μm) is used instead of a normal grating (see Supplementary Information Section 1 and 2). In measurements, unpolarized white light is used to illuminate the sample (Fig. 1b) via a dark-field condenser. The scattered light is collected by an objective (×40, N.A. = 0.6) forming an intermediate image and a final image at the entrance of the spectrometer and camera (iXon Ultra 897, Andor, Belfast, Northern Ireland), respectively.

To measure the spectrum of a single nanostructure, first, one needs to take a dark-field image without the LCPG (Fig. 1c) and select the target nanostructure with the help of the slit at the intermediate image plane. Then, the SCD spectrum can be obtained by inserting the LCPG in front of the camera and taking an image. As shown in Fig. 1d, the LCPG separates the LCP and RCP components into opposite orders, thus forming a pair of mirrored strips of spectral lines on the two sides of the 0^th^ order in the image. The difference of the two spectral lines gives the SCD spectrum. In this work, we normalized all the SCD data with the spectrum of the incident light.

It is worth noting that the diffraction efficiencies for the LCP and RCP components are always equal. This is because the polarization-dependent diffractions of the LCPG are caused by the geometric phases of the LCP and RCP light which are always conjugated with each other^26,31^. The detailed discussion can be found in the Supplementary Information of this work.

### Scattering CD spectroscopy of single vertically-coupled Au nanorod pair

To test the performance of the SCD spectroscopy system, vertically-coupled Au nanorod pairs were used as the standard sample in this work. The sample was fabricated using the two-step electron-beam lithography technique. As shown in Fig. 1b, each enantiomer consists of two identical Au nanorods (*L* = 140 nm, *W* = 80 nm, *H* = 50 nm) which are not in the same plane and vertically separated by a distance of 50 nm (i.e., *D* = 100 nm). And the helical arrangement of the Au nanorod pair in the two enantiomers are mirror images of each other, i.e., the right-hand enantiomer is dextrogyrate while the left-hand enantiomer is laevogyrate.

Figure 1e and f are the SCD spectra (green solid line) of a nanorod pair and its enantiomer, respectively. The SCD spectra exhibit a typical bisignate CD line shape which possesses both a positive band and a negative band. For the right-hand enantiomer, a negative band emerges first with a positive band following subsequently; while the SCD spectrum of the left-hand enantiomer is inverse to its mirror symmetric structure.

**Fig. 2:**
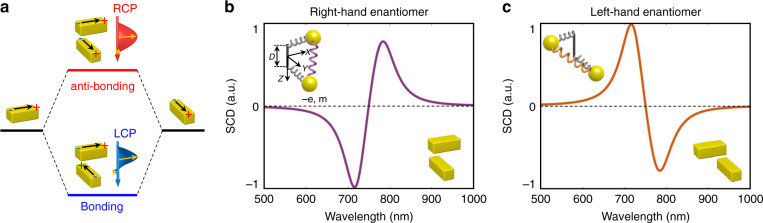
Origin of the bisignate SCD spectral profile. **a** The hybridization theory for the bisignate spectrum. **b**, **c** Are the SCD spectra of a right-hand and left-hand nanorod pair predicted by the Born–Kuhn model, respectively

The results are similar to the case of transmission CD spectra collected from the array samples^8^ and can be pictorially understood by considering the hybridization theory as depicted in Fig. 2a. Due to the coupling, the resonance modes of the two nanorods form two different hybrid modes, bonding mode and anti-bonding mode^32,33^. When a nanorod pair, e.g., right-hand enantiomer, is illuminated by natural polarized light, both the bonding mode and anti-bonding mode will be excited, and they will scatter light into LCP and RCP components, respectively. Note that the energy of bonding mode is lower than the anti-bonding mode. Therefore, the resonance wavelength of the scattering spectrum of the RCP light component is shorter than that of the LCP light component, and the SCD spectrum (*i.e*., *I*_*SCD*_ = *I*_*LCP*_ − *I*_*RCP*_) has a negative peak on the blue side and a positive peak on the red side. In the case of left-hand enantiomers, the signs of the peaks are opposite. This is exactly what we observed in Fig. 1e and f.

### Theory for the polarization-dispersive imaging spectrometer

The above coupling induced optochiral effects can also be explicitly described using the coupled oscillator model, i.e., the Born–Kuhn model^8,34^, which is an extended version of the Lorentz bound electron model^35^. As shown in Fig. 2b, the model consists of two electrons (charge -*e* and mass *m*) bounded to two perpendicularly arranged elastic springs, and the dynamics of the system under external electric fields can then be described as

1 $$\left\{ {\begin{array}{*{20}{c}} {\ddot x + \gamma \dot x + \omega _0^2x + \xi y = - \frac{e}{m}E_xe^{ - i\omega t - ik\frac{D}{2}}} \\ {\ddot y + \gamma \dot y + \omega _0^2y + \xi x = - \frac{e}{m}E_ye^{ - i\omega t + ik\frac{D}{2}}} \end{array}} \right.$$

Here, *γ* is the damping parameter, *ω*_0_ is the natural resonant frequency of the oscillator, *ξ* is the coupling strength of the two oscillators, and *D* is the vertical separation between them. We can obtain the time varying displacement **l** of the oscillators by solving Eq. (1) and derive the dipole moment using **P** = *q***l**. The results of the dipole moment in two mutually orthogonal directions are

2-1 $$P_x\left( {{{{\mathbf{r}}}},t} \right) = \frac{{e^2}}{m}\left[ {\frac{{\omega _0^2 - \omega ^2 - i\gamma \omega }}{{(\omega _0^2 - \omega ^2 - i\gamma \omega )^2 - \xi ^2}}E_x + } \right.\left. {\frac{{ - \xi }}{{(\omega _0^2 - \omega ^2 - i\gamma \omega )^2 - \xi ^2}}E_ye^{ikD}} \right]e^{i\left( {{{{\mathbf{k}}}} \cdot {{{\mathbf{r}}}} - \omega t} \right)}$$

2-2 $$P_y\left( {{{{\mathbf{r}}}},t} \right) = \frac{{e^2}}{m}\left[ {\frac{{ - \xi }}{{(\omega _0^2 - \omega ^2 - i\gamma \omega )^2 - \xi ^2}}E_xe^{ - ikD} + } \right.\left. {\frac{{\omega _0^2 - \omega ^2 - i\gamma \omega }}{{(\omega _0^2 - \omega ^2 - i\gamma \omega )^2 - \xi ^2}}E_y} \right]e^{i\left( {{{{\mathbf{k}}}} \cdot {{{\mathbf{r}}}} - \omega t} \right)}$$

Considering *e*^±*ikD*^ ≈ 1 ± *ikD*, Eq. (2) can be rewritten into a simple form:

3 $$\left( {\begin{array}{*{20}{c}} {P_x} \\ {P_y} \end{array}} \right) = \varepsilon _0\left( {\begin{array}{*{20}{c}} {\alpha _{xx}} & {\alpha _{xy} + ik{\Gamma}} \\ {\alpha _{yx} - ik{\Gamma}} & {\alpha _{yy}} \end{array}} \right)\left( {\begin{array}{*{20}{c}} {E_x} \\ {E_y} \end{array}} \right)e^{i\left( {{{{\mathbf{k}}}} \cdot {{{\mathbf{r}}}} - \omega t} \right)}$$

Here, Г is known as the nonlocal optical susceptibility^34,36,37^ and has the expression:

4 $${\it{{\Gamma}}} = \frac{{De^2}}{{\varepsilon _0m}}\frac{{ - \xi }}{{(\omega _0^2 - \omega ^2 - i\gamma \omega )^2 - \xi ^2}}$$

In our experiment, scattered light *I*_*sca*_ = |**E**_sca_ | ^2^ is measured. Here, **E**_sca_ is linearly related to the factor of *ω*^2^**P**^38^. We can then connect the SCD signal, which is the difference of the LCP and RCP components of **E**_sca_ to the dipole moment **P**.

5 $$I_{SCD} = I_{sca,L} - I_{sca,R} \approx \omega ^4\left| {{{{\mathbf{P}}}}_L} \right|^2 - \omega ^4\left| {{{{\mathbf{P}}}}_R} \right|^2$$

Since the illumination light is unpolarized, the SCD signal is the average of the scattered light induced by the excitation field along all the angle *θ*.

6 $$I_{SCD} \propto \frac{{\omega ^4}}{{2\pi }}{\int}_0^{2\pi } {\left( {\left| {{{{\mathbf{P}}}}_L} \right|^2 - \left| {{{{\mathbf{P}}}}_R} \right|^2} \right)\;} d\theta$$

Here, **P**_L_ and **P**_R_ are the LCP and RCP components of **P**, respectively. They can be calculated by using the point product of **P** and the basis vectors of the LCP and RCP light components in our laboratory coordinate frame, *i.e*., $${{{\mathbf{e}}}}_{L,R} = \frac{{\sqrt 2 }}{2}\left( {\begin{array}{*{20}{c}} 1 \\ { \pm i} \end{array}} \right)$$. The detailed expression can be derived using the Born–Kuhn model and is given by

7 $$I_{{SCD}_{LH}^{RH}} \approx A\frac{{\pm\xi \left({\omega _0^2\omega ^5 - \omega ^7} \right)}}{{\left( {\omega _0^4 - 2\omega _0^2\omega ^2 + \omega ^4 - \gamma ^2\omega ^2 - \xi ^2} \right)^2 + \left({2\gamma \omega ^3 - 2\gamma \omega _0^2\omega} \right)^2}}$$

for right-hand and left-hand enantiomers of the nanorod pairs. The notation *ξ* is always a positive number in Eq. (7), and *A* is a positive constant coefficient. Detailed calculations can be found in Supplementary Information. Comparing Eq. (7) and Eq. (4), we know

8 $$I_{SCD} \propto \omega ^4Im\left( { - {\it{{\Gamma}}}} \right)$$

From Eq. (8), we learn that the profile of a SCD spectrum is determined by the parameters of the coupled oscillators, *ω*_0_, *γ* and *ξ*. Here, *ω*_0_, *γ* and *ξ* correspond to the wavelength of the zero-value point, linewidth and size of the splitting between the two bisignate peaks, respectively. As illustrated in Fig. 2b and c, we substitute parameters *γ* = 250 THz, *ω*_0_ = 2512 THz, and *ξ* = 5 × 10^29^ s^−2^ into Eq. (7) to obtain a typical set of SCD spectra for the right-hand and left-hand enantiomers, and the calculated results are similar to the experimental SCD spectra shown in Fig. 1e and f.

### Spectral interpretation for coupled Au nanorod pairs

We can further fit the experimental data with the Eq. (8), and retrieve the key parameters such as *γ*, *ω*_0,_ and *ξ* of a structure. For example, in the case of the result in Fig. 1e, we have *γ* = 250 THz, *ω*_0_ = 2472 THz, and *ξ* = 5.5 × 10^29^ s^−2^; in Fig. 1f, we have *γ* = 250 THz, *ω*_0_ = 2552 THz, and *ξ* = 5 × 10^29^ s^−2^. The fitting results (dashed lines in Fig. 1e and f) match the experimental data almost perfectly. This justifies the Born–Kuhn model above.

**Fig. 3:**
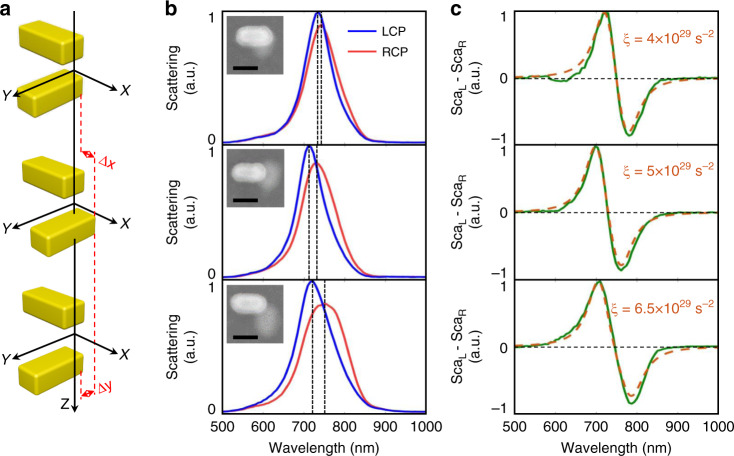
Geometry-dependent SCD spectra. **a** Structure illustration of the vertically coupled nanorod pairs with different displacements. **b** The left-hand and right-hand circularly polarized components of the scattered spectra of the nanorod pairs. The insets are the correlated SEM images. Scale bar: 100 nm. **c** The SCD spectra of the samples. The dashed lines are the fitting results based on the Born–Kuhn model

Since the coupling between the nanorods, *ξ*, is dependent on the relative position of the two nanorods, we can further correlate the spectral information with the geometrical information of the chiral nanostructures. Figure 3 shows the example of the SCD spectra of nanorod pairs with different relative positions. The splitting of the two bisignate peaks becomes wider when the displacement of the central position of the two nanorods increases. This is because the electric fields are localized at the ends of the nanorods, and when the displacement is larger the ends of the two nanorods will become closer to each other and the coupling strength will increase.

Another interesting point is that, Eq. (8) is very similar to the transmission CD spectrum of the bulk material composed of such coupled oscillators described by the Born–Kuhn model. In the case of bulk model, the CD spectrum can be described as (see Supplementary Information Section 4)

9 $$I_{CD} = I_{abs,L}--I_{abs,R} = \frac{{2\omega ^2}}{{c^2}}Im\left( { - {\it{{\Gamma}}}^\prime } \right)$$

Here, ${\it{{\Gamma}}}^\prime = N{\it{{\Gamma}}}$ is the nonlocal optical susceptibility of the bulk material, and *N* is the density of the oscillators. Both methods describe the spectral profile of the nonlocal susceptibility of the nanostructure (or the bulk material made of such a nanostructure).

Meanwhile, there is also a fundamental difference between *I*_*SCD*_ and *I*_*CD*_, that leads to their different dependences on frequency, *ω*. The SCD spectroscopy measures scattering signals, |**E**_sca_| ^2^; the conventional transmission CD spectroscopy measures the extinction signal, i.e. the cross-term between the scattering and incident fields, |**E**_inc_| |**E**_sca_| ^39^. Here, |**E**_sca_| is proportional to *ω*^2^, while |**E**_inc_| is independent to *ω*. As a result, $I_{SCD} \propto \omega ^4$, while $I_{CD} \propto \omega ^2$.

### Scattering CD spectroscopy of single 3D DNA-assembled nanostructures

After establishing the relation between the spectral profile and the structural information, we can apply this LCPG-based SCD spectroscopy system to more complex nanostructures. In this work, we focus on 3D DNA-assembled plasmonic nanostructures, which often exhibit rich optical behaviors due to the geometrical variations at the single nanostructure level.

**Fig. 4:**
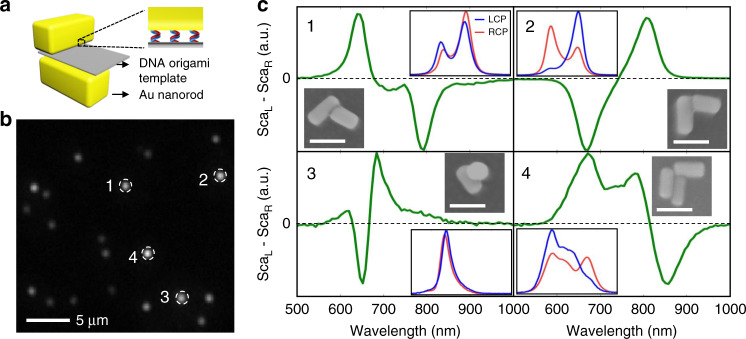
SCD spectroscopy of single DNA-assembled 3D nanostructures. **a** Schematic drawing of the 3D DNA-assembled Au nanorod pairs. **b** The dark-field image of the sample. **c** The SCD spectra of the spots labeled in (**b**) and the correlated SEM images. The blue and red lines in the insets are the left-hand and right-hand circularly polarized components of the scattering spectra of the structures, respectively. Scale bar: 100 nm

More detailed, we assembled Au nanorods into vertically coupled pairs by using a ribbon-like DNA origami nanostructure as the template^40,41^, as shown in Fig. 4a. In the measurements, a dark-field image was first taken without the LCPG (Fig. 4b), then the target nanostructures (labeled by dashed circles) were chosen and measured with the LCPG in the system. In addition, SEM images of the same sample were also collected. After comparing the optical images with the SEM images, we are able to correlate the SCD signals of each individual nanostructure with its SEM images directly, as shown in Fig. 4c.

Here, we first checked the nanorod pairs, which consist of two stacked horizontal nanorods, as shown in Fig. 4c1–2. Symmetric bisignate SCD spectra are observed similar to the nanofabricated nanorod pairs in Fig. 1. Interestingly, the splitting between the two peaks of the DNA-assembled nanorod pairs can reach 150 nm, significantly larger than the case of nanofabricated nanorod pairs. This can be explained by considering that the spacing in the DNA-assembled structures is much smaller than the nanofabricated ones and consequently the inter-nanorod coupling is much stronger.

Another intriguing point is that the signs of the SCD spectra in Fig. 4c1 and Fig. 4c2 are opposite to each other, indicating that the two structures have opposite handedness. However, one can hardly distinguish this 3D structural difference using the SEM images (insets of Fig. 4c1–2). This directly demonstrates the power of SCD spectroscopy for studying the conformation of complex 3D chiral nanostructures.

In the same sample, other types of DNA-assembled nanorod structures were also found. For example, Fig. 4c3 shows the result of a nanorod pair consisting of a horizontal nanorod and a vertical one. In this case, bisignate SCD spectrum is still observed, but the spectral profile is different from the structures made of two horizontal nanorods due to the different structure orientation. In addition, we also found a nanorod trimer in the same sample area (Fig. 4c4), and multiple resonances were observed instead of two peaks. This can be attributed to the multiple coupling channels between the three nanorods. The above results show that, the SCD spectra are not only sensitive to the handedness, but also related to the orientation and detailed components of a nanostructure. It is, therefore, interesting to generalize the Bohn–Kuhn model for more complex situations. This will potentially allow researchers to retrieve all the detailed 3D structural information in the nanoscale by taking a SCD spectrum.

## Discussion

Finally, we would like to emphasize that light scattering is a complex process. The polarization of the scattered fields is directly related to the polarization of the incident. To obtain a spectrum corresponding to the case of conventional CD measurements, unpolarized incident (i.e., its Stokes parameters satisfy *S*_*1*_ = *S*_*2*_ = *S*_*3*_ = 0) is required in the polarization-dispersive imaging spectrometer developed in this work (see Supplementary Information Section 3). If polarized or partially polarized light is used, the result can be significantly different depending on the polarization state of the incident.

In summary, we developed a new type of LCPG-based polarization-dispersive imaging spectrometer for measuring the scattering CD spectra of single nanostructures. This method is simple, fast, and explicit in data interpretation. In practice, it can be easily realized by adding a LCPG into a conventional dark-field microscope; the SCD spectrum of a single nanostructure can be obtained with a single shot without modulating the polarization state of the incident light; the measured data is directly proportional to the imaginary part of the nonlocal susceptibility of the nanostructure, corresponding to the case of conventional transmission CD spectroscopy for bulk materials. With the help of this technique, we measured the SCD spectra of single DNA-assembled plasmonic chiral nanostructures and further correlated the spectra with their geometry using SEM images of the same structures. Rich optical responses were observed from the individual nanostructures, and it shows the power and versatility of the LCPG-based SCD spectroscopy method for investigating individual complex 3D chiroptical nanostructures.

## Materials and methods

### Fabrication of the vertically-coupled Au nanorod pairs

The vertically-coupled Au nanorod pair structure was fabricated using a two-step electron-beam lithography technique. The gold film (50 nm thick) was deposited first by a evaporator on the glass substrate coated with indium tin oxide (ITO) thin film. And the first electron-beam lithography step was finished with the help of the spin-coated negative photoresist, following by the inductively coupled plasma etching process to obtain the lower nanorod. Then, UV curing adhesive was spincasted on the top as a spacer layer. After that, the second electron-beam lithography step was done to fabricate the upper nanorod, which is the same as the first step. The alignment of the nanorods was achieved with the help of marks fabricated in the first step.

### Liquid crystal polarization grating

The LCPG was homemade using the commercial nematic LC E7 purchased from Jiangsu Hecheng Display Technology Co., Ltd. The LC directors of LCPG were guided by a thin photoalignment layer preprogramed using a dynamic-mask photopatterning technique^42^. They are periodically arranged in the sample plane with a distribution function of *α*(*x, y*) = −π*x*/*Λ*, where *Λ* is the period of the polarization grating, having *Λ* = 18 μm (see Supplementary Information Section 2).

## Supplementary information

Supplementary Information for Polarization-Dispersive Imaging Spectrometer for Scattering Circular Dichroism Spectroscopy of Single Chiral Nanostructures
