# Supplementary material for: Polarization-dispersive imaging spectrometer for scattering circular dichroism spectroscopy of single chiral nanostructures
Source: Light Sci Appl. 2022 Mar 18;11:64. doi: 10.1038/s41377-022-00755-2 (PMC8933428; doi:10.1038/s41377-022-00755-2)
Supplement: Supplementary file 1 — Supplementary Information for Polarization-Dispersive Imaging Spectrometer for Scattering Circular Dichroism Spectroscopy of Single Chiral Nanostructures [file 41377_2022_755_MOESM1_ESM.docx]

Supplementary Information for

Polarization-Dispersive Imaging Spectrometer for Scattering Circular Dichroism Spectroscopy of Single Chiral Nanostructures

*Shuang Zhou^1^, Jie Bian^1,2^*, Peng Chen^1^, Mo Xie^3^, Jie Chao^3^, Wei Hu^1^, Yanqing Lu^1^ and Weihua Zhang^1,2^**

1, College of Engineering and Applied Sciences, MOE Key laboratory of Intelligent Optical Sensing and Manipulation, Nanjing university, Nanjing 210023, China.

2, State Key Laboratory of Analytical Chemistry for Life Science, and Jiangsu Key Laboratory of Artificial Functional Materials, Nanjing University, Nanjing 210093, China.

3, Key Laboratory for Organic Electronics and Information Displays (KLOEID) & Jiangsu Key Laboratory for Biosensors, Institute of Advanced Materials (IAM), National Synergetic Innovation Center for Advanced Materials (SICAM), Nanjing University of Posts and Telecommunications, 9 Wenyuan Road, Nanjing 210023, China.

**1. The polarization-dispersive imaging spectrometer**

Figure S1 illustrates the structure of the polarization-dispersive imaging spectrometer, which was attached on the side port of the microscope. The spectrometer consists of a slit, a pair of lenses, a LCPG and a CCD camera. The focal lengths of the two lenses are 15 cm and 30 cm respectively.

Fig. S1 Schematic drawing of the polarization-dispersive imaging spectrometer.

In the imaging mode, the slit is fully open and the LCPG is flipped down allowing us to take normal microscopic images. In the spectral mode, the slit size is tuned to approximately 80 µm, and the LCPG is flipped up diffracting optical signals to different locations of the CCD depending on their polarization and wavelength.

**2. The liquid crystal polarization grating**

A homemade LCPG was used in this work with commercial nematic liquid crystal (LC E7, Jiangsu Hecheng Display Technology Co., Ltd. China). It is a thin (5 μm thick) LC filled cell formed by the two parallel ITO coated glasses. The phase retardation, *ψ*, can be tuned by applying voltages between the two ITO electrodes.

Fig. S2 Polarization-dependent light dispersion by the liquid crystal polarization grating.

To form the grating, one of the ITO glass was coated by a director molecule layer, in which the orientation of the director molecules, consequently the local orientation of the LC molecules, *α*, vary continuously along one lateral direction forming a periodic structure with a pitch size of 18 μm. This structure induces conjugated space-variant geometric phases for LCP and RCP light, forming the ±1th order of diffractions with efficiency^s1^

$\eta_{\pm1}=\frac{1\pm S_{3}}{2}{sin}^{2}\frac{\psi}{2}$ (s1).

Here, *S_3_* is the normalized Stokes parameter denoting the fraction of the circular component in the incident. For RCP, *S_3_* = +1, $\eta_{+1}=1$ and $\eta_{-1}=0$; for LCP, *S_3_* = -1, $\eta_{+1}=0$ and $\eta_{-1}=1$. It is evident that the RCP and LCP components of the light are always split into the ±1th order of diffractions with equal efficiency.

The LCPG was rigorously tested before being used in CD measurements. Its total transmission efficiency is 83%, and the diffraction efficiency is 92.8% at 671 nm (driving voltage 4.5 V, frequency 1kHz).

To calibrate the spectrometer, we recorded positions, $x_{\pm1}\left( \lambda\right)$, of the diffraction spots of lasers at different wavelengths (405 nm, 532 nm, 671 nm and 785 nm) and calculated the corresponding wavelength for each pixel using linear interpolation. The data collected by the camera, $I\left( x \right)$ can then be converted to $I_{\pm1}\left( \lambda\right)$. To remove the influence of the spectral profile of the incident light *I_inc_*, all the SCD spectra were normalized in this work,

$I_{\mathrm{SCD}}\left( \lambda\right)=\frac{{I_{-1}\left( \lambda\right)-I}_{+1}\left( \lambda\right)}{I_{\mathrm{inc}}\left( \lambda\right)}$ (s2)

**3. The SCD signal of a single Au nanorod pair by Born-Kuhn model**

The optical activity originating from the vertically-coupled Au nanorod pair can be described by Born-Kuhn model.^s2^ The model consists of two electrons (charge -e and mass m) bounded to two perpendicularly arranged elastic springs. In our laboratory coordinate frame, the equilibrium position of the upper nanorod is set as **r**_0_*_x_* = (*x_0_*, *y_0_*, -*D*/2), while the lower one is **r**_0_*_y_* = (*x_0_*, *y_0_*, *D*/2). And the dynamics of the system under external electric fields can then be described as

$\left\{ \begin{aligned} \ddot{x}+\gamma\dot{x}+{\omega_{0}}^{2}x+\xi y=-\frac{e}{m}E_{x}e^{-i\omega t-ik\frac{D}{2}} \\ \ddot{y}+\gamma\dot{y}+{\omega_{0}}^{2}y+\xi x=-\frac{e}{m}E_{y}e^{-i\omega t+ik\frac{D}{2}} \end{aligned} \right.$ (s3)

Here, *γ* is the damping parameter, *ω*_0_ is the natural resonant frequency of the oscillator, *ξ* is the coupling strength of the two oscillators, and *D* is the vertical separation between them. We substitute the ansatzes *x*(*t*) = *x_0_e*^-^*^iωt^* and *y*(*t*) = *y_0_e*^-^*^iωt^* into eq. s3, and the time vary displacement **l** of the oscillators in two mutual orthogonal directions are given by

$x\left( \mathbf{r}_{0x},t \right)=-\frac{e}{m}\left[ \frac{{\omega_{0}}^{2}-\omega^{2}-i\gamma\omega}{({\omega_{0}}^{2}-\omega^{2}-i\gamma\omega)^{2}-\xi^{2}}E_{x}+ \right.\left. \frac{-\xi}{({\omega_{0}}^{2}-\omega^{2}-i\gamma\omega)^{2}-\xi^{2}}E_{y}e^{ikD} \right]e^{-i\omega t-ik\frac{D}{2}}$ (s4-1)

$y\left( \mathbf{r}_{0y},t \right)=-\frac{e}{m}\left[ \frac{-\xi}{({\omega_{0}}^{2}-\omega^{2}-i\gamma\omega)^{2}-\xi^{2}}E_{x}e^{-ikD}+ \right.\left. \frac{{\omega_{0}}^{2}-\omega^{2}-i\gamma\omega}{({\omega_{0}}^{2}-\omega^{2}-i\gamma\omega)^{2}-\xi^{2}}E_{y} \right]e^{-i\omega t+ik\frac{D}{2}}$ (s4-2)

The dipole moment can then be obtained using **P** = *q***l** . The results are

$P_{x}\left( \mathbf{r},t \right)=\frac{e^{2}}{m}\left[ \frac{{\omega_{0}}^{2}-\omega^{2}-i\gamma\omega}{({\omega_{0}}^{2}-\omega^{2}-i\gamma\omega)^{2}-\xi^{2}}E_{x}+ \right.\left. \frac{-\xi}{({\omega_{0}}^{2}-\omega^{2}-i\gamma\omega)^{2}-\xi^{2}}E_{y}e^{ikD} \right]e^{i\left( \boldsymbol{k\cdot r}-\omega t \right)}$ (s5-1)

$P_{y}\left( \mathbf{r},t \right)=\frac{e^{2}}{m}\left[ \frac{-\xi}{({\omega_{0}}^{2}-\omega^{2}-i\gamma\omega)^{2}-\xi^{2}}E_{x}e^{-ikD}+ \right.\left. \frac{{\omega_{0}}^{2}-\omega^{2}-i\gamma\omega}{({\omega_{0}}^{2}-\omega^{2}-i\gamma\omega)^{2}-\xi^{2}}E_{y} \right]e^{i\left( \boldsymbol{k\cdot r}-\omega t \right)}$ (s5-2)

We can expand *e*^±^*^ikD^* ≈ 1 ± *ikD …* In this case, the first two terms are retained. The second term indicates that the optical response is nonlocal by considering the finite size of the nanorod pair in which the oscillators are spatially separated. And the optical effect correlated linearly with the wave vector is referred to as first-order spatial dispersion phenomenon, *i.e.* optical activity. As a result, eq. s5 can be rewritten into a simple form:

$\left( \begin{matrix} P_{x} \\ P_{y} \end{matrix} \right)\mathbf{=}\varepsilon_{0}\left( \begin{matrix} \alpha_{xx} & \alpha_{xy}+ik\Gamma\\ \alpha_{yx}-ik\Gamma& \alpha_{yy} \end{matrix} \right)\left( \begin{matrix} E_{x} \\ E_{y} \end{matrix} \right)e^{i\left( \boldsymbol{k\cdot r}-\omega t \right)}$ (s6)

Here, *α* is the optical susceptibility, and *Г* is known as the nonlocal optical susceptibility. The expressions are:

$\alpha_{xx}=\alpha_{yy}=\frac{e^{2}}{\varepsilon_{0}m} \frac{{\omega_{0}}^{2}-\omega^{2}-i\gamma\omega}{({\omega_{0}}^{2}-\omega^{2}-i\gamma\omega)^{2}-\xi^{2}}$ (s7-1)

$\alpha_{xy}=\alpha_{yx}=-\frac{e^{2}}{\varepsilon_{0}m} \frac{\xi}{({\omega_{0}}^{2}-\omega^{2}-i\gamma\omega)^{2}-\xi^{2}}$ (s7-2)

$\Gamma=\frac{De^{2}}{\varepsilon_{0}m} \frac{-\xi}{({\omega_{0}}^{2}-\omega^{2}-i\gamma\omega)^{2}-\xi^{2}}$ (s7-3)

In our experiment, scattered light *I_sca_* = |**E**_sca_|^2^ is measured. The scattering field **E**_sca_ can be described by introducing the Green function: ^s3^

$\mathbf{E}_{sca}\left( \mathbf{r} \right)=\omega^{2}\mu_{0}\mu\mathbf{G}\left( \mathbf{r},\mathbf{r}_{0} \right)\mathbf{P}\propto\omega^{2}\mathbf{P}$ (s8)

Here, **E**_sca_ is linearly related to the factor of *ω*^2^**P**. We can then connect the SCD signal which is the difference of the LCP and RCP components of **E**_sca_ to the dipole moment **P**.

$I_{SCD}=I_{sca,L}-I_{sca,R}\approx{\omega^{4}\left| \mathbf{P}_{L} \right|}^{2}-\omega^{4}\left| \mathbf{P}_{R} \right|^{2}$ (s9)

Since the illumination light is unpolarized, the SCD signal is the average of the scattered light induced by the excitation field along all the angle *θ*.

$I_{SCD}\propto\frac{\omega^{4}}{2\pi}\int_{0}^{2\pi} \left( \left| \mathbf{P}_{L} \right|^{2}-\left| \mathbf{P}_{R} \right|^{2} \right)d\theta$ (s10)

Here, **P**_L_ and **P**_R_ are the LCP and RCP components of **P**, respectively. They can be calculated by using the point product of **P** and the basis vectors of the LCP and RCP light components in our laboratory coordinate frame, *i.e.*, $\mathbf{e}_{L,R}=\frac{\sqrt{2}}{2}\left( \begin{matrix} 1 \\ \pm i \end{matrix} \right)$. We substitute the excitation field with the polarization angle of *θ,* which can be written as $\mathbf{E}_{0}=\left( \begin{matrix} cos\theta\\ sin\theta\end{matrix} \right)$, into eq. s6. The result is

$\mathbf{P}_{L}=\frac{\sqrt{2}}{2}\left( \begin{matrix} C_{L} \\ iC_{L} \end{matrix} \right)$,$\mathbf{P}_{R}=\frac{\sqrt{2}}{2}\left( \begin{matrix} C_{R} \\ -iC_{R} \end{matrix} \right)$ (s11)

where *C_L_* and *C_R_* are the coefficients and have the expressions:

$C_{L}=\frac{\sqrt{2}}{2}\varepsilon_{0}\left[ \alpha_{xx}cos\theta+\alpha_{xy}sin\theta-k\Gamma cos\theta\right.+\left. ik\Gamma sin\theta-i\alpha_{yx}cos\theta-i\alpha_{yy}sin\theta\right]$ (s12-1)

$C_{R}=\frac{\sqrt{2}}{2}\varepsilon_{0}\left[ \alpha_{xx}cos\theta+\alpha_{xy}sin\theta+k\Gamma cos\theta\right.+\left. ik\Gamma sin\theta+i\alpha_{yx}cos\theta+i\alpha_{yy}sin\theta\right]$ (s12-2)

The SCD signal can be further written as:

$I_{SCD}\propto\frac{\omega^{4}}{2\pi}\int_{0}^{2\pi} \left( \left| \mathbf{P}_{L} \right|^{2}-\left| \mathbf{P}_{R} \right|^{2} \right)d\theta=\frac{\omega^{4}}{2\pi}\int_{0}^{2\pi} \left( \left| C_{L} \right|^{2}-\left| C_{R} \right|^{2} \right)d\theta$ (s13)

We substitute eq. s7 and eq.s12 into eq. s13, and then obtain the SCD signal:

$I_{SCD}\approx A\frac{\xi\left( \omega_{0}^{2}\omega^{5}-\omega^{7} \right)}{\left( \omega_{0}^{4}-2\omega_{0}^{2}\omega^{2}+\omega^{4}-\gamma^{2}\omega^{2}-\xi^{2} \right)^{2}+\left( 2\gamma\omega^{3}-2\gamma\omega_{0}^{2}\omega\right)^{2}}$ (s14)

where *A* is a positive constant coefficient.

In our laboratory coordinate frame, the coupling parameter of the right-hand enantiomers has the relationship as *ξ* > 0; while for the left-hand enantiomers, the relationship is *ξ* < 0. The final expression of the SCD signal is

${I_{SCD}}_{LH}^{RH}\approx A\frac{\pm\xi\left( \omega_{0}^{2}\omega^{5}-\omega^{7} \right)}{\left( \omega_{0}^{4}-2\omega_{0}^{2}\omega^{2}+\omega^{4}-\gamma^{2}\omega^{2}-\xi^{2} \right)^{2}+\left( 2\gamma\omega^{3}-2\gamma\omega_{0}^{2}\omega\right)^{2}}$ (s15)

for right-hand and left-hand enantiomers of the nanorod pairs. The notation *ξ* is always a positive number in eq. s15.

**4. The CD signal of the bulk material**

Optical activity of the chiral material can be derived from the first-order spatial dispersion of the time harmonic electromagnetic field. And the related constitutive equation can be written as

$\mathbf{D}=\varepsilon_{0}\varepsilon_{r}\mathbf{E}+\varepsilon_{0}\Gamma\nabla\mathbf{E=}\varepsilon\mathbf{E}$ (s16)

$\varepsilon_{ij}\left( \omega,\mathbf{k} \right)=\varepsilon_{0}{\varepsilon_{r}}_{ij}\left( \omega\right)+i\varepsilon_{0}\Gamma_{ijl}(\omega)k_{l}$ (s17)

where *Г* is the nonlocal optical susceptibility, characterizing the optical activity of the chiral media.

According to the Fresnel’s hypothesis that the optical activity arises from the difference in the refractive indexes of the RCP and LCP light, we then solve the Helmholtz equation to obtain the eigen-indexes of refraction, just considering the isotropic media which consist of randomly oriented molecules for simplify.

In the principal coordinate system of the chiral isotropic media, the permittivity *ε_r_* and the nonlocal optical susceptibility *Г* have the expressions as *ε_rij_*(*ω*) = *ε_r_δ_ij_* and *Г_ijl_*(*ω*) *= Гe_ijl_*. Here, *e_ijl_* is unit antisymmetric tensor, *i.e.*, *e_xyz_* = *e_zxy_* = -*e_xzy_* = … = 1, *e_iij_* = 0. We substitute the above expressions into Helmholtz equation **k** × (**k** × **E**) + *ω^2^με***E** = 0, and have:

$\left( \begin{matrix} \omega^{2}\mu\varepsilon_{0}\varepsilon_{r}-k_{y}^{2}-k_{z}^{2} & k_{x}k_{y}+i\omega^{2}\mu\varepsilon_{0}\Gamma k_{z} & k_{x}k_{z}-i\omega^{2}\mu\varepsilon_{0}\Gamma k_{y} \\ k_{y}k_{x}-i\omega^{2}\mu\varepsilon_{0}\Gamma k_{z} & \omega^{2}\mu\varepsilon_{0}\varepsilon_{r}-k_{x}^{2}-k_{z}^{2} & k_{y}k_{z}+i\omega^{2}\mu\varepsilon_{0}\Gamma k_{x} \\ k_{z}k_{x}+i\omega^{2}\mu\varepsilon_{0}\Gamma k_{y} & k_{z}k_{y}-i\omega^{2}\mu\varepsilon_{0}\Gamma k_{x} & \omega^{2}\mu\varepsilon_{0}\varepsilon_{r}-k_{x}^{2}-k_{y}^{2} \end{matrix} \right)\left( \begin{matrix} E_{x} \\ E_{y} \\ E_{z} \end{matrix} \right)=0$ (s18)

Considering the incident field propagates along the +Z axis, eq. s18 can be simplified as

$\left( \begin{matrix} \varepsilon_{r}-n^{2} & i\Gamma k \\ -i\Gamma k & \varepsilon_{r}-n^{2} \end{matrix} \right)\left( \begin{matrix} E_{x} \\ E_{y} \end{matrix} \right)=0$ (s19)

And the resulted eigen-vectors and eigen-indexes of refraction are

$E_{L,R}=E_{x}\pm iE_{y}$ (s20-1)

$n_{L,R}^{2}=\varepsilon_{r}\mp\Gamma\frac{\omega}{c}n_{L,R}$ (s20-2)

From eq. s20-2, we can obtain

$\Delta n=n_{L}-n_{R}=\frac{n_{L}^{2}-n_{R}^{2}}{n_{L}+n_{R}}=-\Gamma\frac{\omega}{c}$ (s21)

Combined with the absorption law described by Beer,^s4^ we can get the final expression of the circular dichroism which is given by

$I_{CD}=I_{abs,L}-I_{abs,R}=\frac{2\omega}{c}Im\left( \Delta n \right)=\frac{2\omega^{2}}{c^{2}}Im\left( -\Gamma\right)$ (s22)

**References:**

(s1) Duan, W. *et al.* Fast-response and high-efficiency optical switch based on dual-frequency liquid crystal polarization grating. *Opt. Mater. Express* **6**, 597-602 (2016).

(s2) Yin, X.; Schäferling, M.; Metzger, B.; Giessen, H. Interpreting chiral nanophotonic spectra: The plasmonic born–kuhn model. *Nano Lett.* **2013**, *13* (12), 6238-6243.

(s3) Novotny, L.; Hecht, B. *Principles of nano-optics*, 2nd ed.; Cambridge University press: New York, 2012.

(s4) Fox, M. *Optical properties of solids*; Oxford University Press: New York, 2001.
